# The role of rostral prefrontal cortex in prospective memory: A voxel-based lesion study

**DOI:** 10.1016/j.neuropsychologia.2011.02.045

**Published:** 2011-07

**Authors:** Emmanuelle Volle, Gil Gonen-Yaacovi, Angela de Lacy Costello, Sam J. Gilbert, Paul W. Burgess

**Affiliations:** a Institute of Cognitive Neuroscience - UCL (University College London), 17 Queen Square, London WC1N 3AR, UK; b CR-ICM/UPMC/INSERM UMR-S 975, Pitié-Salpétrière Hospital, 47 Boulevard de l’Hôpital, 75013 Paris, France; c Neuropsychology Department, King's College Hospital NHS Foundation Trust, London SE5 9RS, UK

**Keywords:** Human, Lesion study, Neuropsychology, Rostral prefrontal cortex, Prospective memory

## Abstract

Patients with lesions in rostral prefrontal cortex (PFC) often experience problems in everyday-life situations requiring multitasking. A key cognitive component that is critical in multitasking situations is prospective memory, defined as the ability to carry out an intended action after a delay period filled with unrelated activity. The few functional imaging studies investigating prospective memory have shown consistent activation in both medial and lateral rostral PFC but also in more posterior prefrontal regions and non-frontal regions. The aim of this study was to determine regions that are necessary for prospective memory performance, using the human lesion approach. We designed an experimental paradigm allowing us to assess time-based (remembering to do something at a particular time) and event-based (remembering to do something in a particular situation) prospective memory, using two types of material, words and pictures. Time estimation tasks and tasks controlling for basic attention, inhibition and multiple instructions processing were also administered. We examined brain-behaviour relationships with a voxelwise lesion method in 45 patients with focal brain lesions and 107 control subjects using this paradigm. The results showed that lesions in the right polar prefrontal region (in Brodmann area 10) were specifically associated with a deficit in time-based prospective memory tasks for both words and pictures. This deficit could not be explained by impairments in basic attention, detection, inhibition or multiple instruction processing, and there was also no deficit in event-based prospective memory conditions. In addition to their prospective memory difficulties, these polar prefrontal patients were significantly impaired in time estimation ability compared to other patients. The same region was found to be involved using both words and pictures, suggesting that right rostral PFC plays a material nonspecific role in prospective memory. This is the first lesion study showing that rostral PFC is crucial for time-based prospective memory. The findings suggest that time-based and event-based prospective memory might be supported at least in part by distinct brain regions. Two particularly plausible explanations for the deficit rest upon a possible role for polar prefrontal structures in supporting in time estimation, and/or in retrieving an intention to act. More broadly, the results are consistent with the view that the deficit of rostral patients in multitasking situations might at least in part be explained by a deficit in prospective memory.

## Highlights

► Prospective memory (PM) is a key cognitive component required in multitasking. ► This lesion study examines the critical regions for event- and time-based PM ► We show that the right rostral prefrontal cortex is necessary for time-based PM. ► Distinct prefrontal regions are associated with deficits in event- and time-based PM. ► The PM deficit of rostral patients might explain their deficit in multitasking situations.

## 1 Introduction

The term “mystery” was used by Mesulam (Mesulam, 1986; see also Burgess, Alderman, Volle, Benoit, & Gilbert, 2009) to describe the behaviour of patients with frontal lobe damage who showed intact performance on traditional neuropsychological assessment of intellectual ability, memory, language, motor skills, perception, and problem-solving, but at the same time showed strong disturbances in everyday life. In the last 20 years, several hypotheses have been proposed in order to explain the problems experienced by these patients (Burgess, 2000; Duncan, Burgess, & Emslie, 1995; Shallice & Burgess, 1991). Burgess, Shallice and collaborators have attempted formal quantification of the difficulties experienced by these patients in everyday life. They designed specific tests to identify a deficit in relatively ill-structured situations (i.e. requiring participants to organise their own behaviour rather than following specific instructions), and identified a specific brain region where damage was associated with these problems: rostral prefrontal cortex (rostral PFC) or frontopolar cortex (Bird, Castelli, Malik, Frith, & Husain, 2004; Burgess, 2000; Burgess et al., 2009; Burgess, Veitch, de Lacy Costello, & Shallice, 2000; Shallice & Burgess, 1991).

More specifically, rostral prefrontal patients may exhibit difficulties when there are several possible ways to behave, when the behaviour is not fully guided by the environment (i.e. what to do and when to act have to be decided by the person), and when two or more tasks have to be engaged alternately, by interleaving. Preparing a meal, or shopping, are typical examples of real-life situations that make these demands. Situations of this type have been labelled as requiring “multitasking” (Burgess et al., 2000). Problems with multitasking can now be assessed with specific neuropsychological tests such as the Six Element Test, the Greenwich test or the Multiple Errands Test (Burgess, Alderman, Evans, Emslie, & Wilson, 1998; Burgess et al., 2000, 2009; Shallice & Burgess, 1991). In a lesion study of 60 patients, using the Greenwich test (Burgess et al., 2000), Burgess and collaborators concluded that rostral patients “*did not do what they intended to do*, despite being able to learn the task rules, form a plan, remember their action, and say what they should have done”. In other words, these patients appeared mainly impaired in the ‘intentional’ component of multitasking. In cognitive psychology, the processes that allow the realisation of an intention after a delay are gathered in the concept of “prospective memory” (Meacham & Dumitru, 1976).

Prospective memory is defined as the ability to carry out a delayed intended action. It refers to a type of memory that allows maintaining and retrieving future plans, goals and activities, which is a crucial ability for human everyday life. Two types of prospective memory can be considered: time-based and event-based (Harris, 1984; Kvavilashvili & Ellis, 1996; for a review see McDaniel & Einstein, 2007). Time-based prospective memory consists of remembering to do something at a particular time, for example remember the meeting with Paul at 5 pm. Event-based prospective memory consists of remembering to do something in a particular situation. For instance, remember to ask Paul for his book next time I meet him.

Experimental testing tries to imitate these real life situations, asking subjects to maintain an intention while doing something else – called the ongoing task – and to retrieve this intention at the appropriate moment, determined either by time or by a given situation.

The few functional imaging studies that have been performed using such tasks (Burgess, Quayle, & Frith, 2001; Burgess, Scott, & Frith, 2003; Gilbert, Gollwitzer, Cohen, Burgess, & Oettingen, 2009; Okuda et al., 1998, 2007; den Ouden, Frith, Frith, & Blakemore, 2005; Reynolds, West, & Braver, 2009; Simons, Scholvinck, Gilbert, Frith, & Burgess, 2006) have shown consistent activation in rostral PFC (in Brodmann area [BA] 10), but also in more posterior prefrontal regions and in non frontal regions. It therefore appears that the rostral PFC is often activated by prospective memory tasks. But are patients with rostral frontal lesions impaired in these tasks?

Functional imaging cannot formally demonstrate whether a region is critical for a task or a function. Lesion studies are thus necessary to indicate for which tasks and processes rostral PFC functioning is necessary. This approach is all the more important because functional imaging studies have shown hemodynamic changes in rostral PFC in many different cognitive paradigms (Christoff & Gabrieli, 2000; Ramnani & Owen, 2004), such as those involving memory retrieval (Simons, Owen, Fletcher, & Burgess, 2005), working memory (Owen, McMillan, Laird, & Bullmore, 2005; Volle et al., 2005), branching and task switching (Braver & Bongiolatti, 2002; Koechlin, Basso, Pietrini, Panzer, & Grafman, 1999; Koechlin & Hyafil, 2007), relational integration (Christoff et al., 2001; Kroger et al., 2002; Reynolds, McDermott, & Braver, 2006), reasoning (Bunge, Wendelken, Badre, & Wagner, 2005; Green, Fugelsang, Kraemer, Shamosh, & Dunbar, 2006; Green, Kraemer, Fugelsang, Gray, & Dunbar, 2010; Volle, Gilbert, Benoit, & Burgess, 2010), and even in simple attention tasks (Pollmann, 2001) or during rest or daydreaming (Buckner, Andrews-Hanna, & Schacter, 2008; Gilbert, Dumontheil, Simons, Frith, & Burgess, 2007; Gusnard & Raichle, 2001; Mason et al., 2007). New techniques for lesion studies, such as voxel-by-voxel lesion-deficit mapping allow precise clinical–radiological correlations by testing all damaged voxels. They do not rely upon classifying patients into categorical groups or choosing a cut-off for pathology, in contrast with more classical methods (Baier et al., 2010; Bates et al., 2003; Dronkers, Wilkins, Van Valin, Redfern, & Jaeger, 2004; Frank, Damasio, & Grabowski, 1997; Gläscher et al., 2009; Kinkingnehun et al., 2007; Tyler, Marslen-Wilson, & Stamatakis, 2005; Rorden & Karnath, 2004; Volle et al., 2008). Instead, statistical tests are performed at each voxel or cluster of voxels, by considering patients damaged in that voxel and comparing them to control values. Because of this voxel-by-voxel testing, and because patients’ lesions are analysed within the same template as fMRI studies, these new lesion methods give results more comparable to functional imaging ones. Yet the lesion approach has rarely been used to explore the cerebral correlates of prospective memory, and there is little evidence showing the critical regions for prospective memory (Burgess et al., 2008).

Therefore, we conducted a lesion study in 45 patients with focal brain lesions, carefully screened for potential confounding cognitive deficits, using a voxel-based method, combined with both time- and event-based prospective memory tasks.

## 2 Materials and methods

The experiment was approved by the local research ethics committee. All participants were able to provide written, witnessed, consent.

### 2.1 Subjects (see Table 1 with patients’ details)

Patients were recruited mainly from the Neurosurgery and the Neurological Departments of King's College Hospital, London, UK. Additional patients were recruited from two other London hospitals: the Regional Neurological and Rehabilitation Unit of the Homerton University Hospital and the Wolfson Rehabilitation Centre, St. George's Healthcare Trust, Wimbledon. Sixty-seven patients were assessed, when attending for a full investigation of their lesion, if they met the following criteria. (i) The presence of a cerebral focal lesion was confirmed by an anatomical CT scan or MRI, available for the current condition. (ii) The lesion was acquired in adulthood (mostly haemorrhage, ischemic stoke or brain tumour; see Table 1). (iii) Participants were able to understand and perform the cognitive tasks. Patients who demonstrated gross disorientation or visual, memory, reading, naming or instrumental impairments that would interfere with the tasks were excluded (impairments detected on VOSP perception battery, on Shortened Revised Token Test (De Renzi & Faglioni, 1978), on the National Adult Reading Test – NART (Bright, Jaldow, & Kopelman, 2002; Nelson & O’Connell, 1978) and on McKenna confrontation naming test (McKenna & Warrington, 1983), on Warrington's recognition memory test (Warrington, 1984)). (iv) Patients had no prior history of neurological or psychiatric disease requiring hospitalisation, of alcohol or other substance abuse, or of developmental problems. (vi) All included patients were right-hand dominant and had English as their first language. It is important to note that every patient who matched the above criteria was included, regardless of the location of the lesion or the pattern of the cognitive deficit. Of the 67 tested, full data including brain scans were eventually available for 45 patients.

**Fig. 1:**
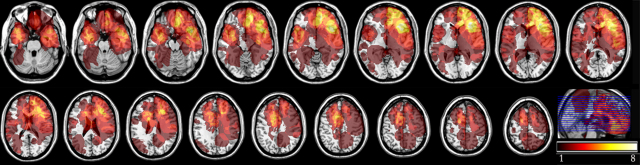
Lesion overlaps of the 45 patients’ lesions, pooled all together. The number of overlapping lesions is represented in warm colours (the lightest, the more overlaps), in the MNI space (according to neurological convention, i.e. right is right).

Fig. 1 shows the location of lesions of these 45 patients. Lesions were located as follows. Twenty patients had a lesion that did not involve the frontal lobes, (i.e. ‘Non Frontal’), but involved temporal (11 patients), parietal (6 patients) and subcortical areas (3 patients). Twenty five patients had a lesion that involved the frontal lobes, among which 8 involved the rostral prefrontal region (‘Rostral PF’; approximately Brodman area 10 [BA10]), and 17 were prefrontal but not rostral (‘Posterior PF’: 6 premotor, 4 dorsolateral prefrontal, 6 ventrolateral and 1 orbitoventral lesions).

Normative data was acquired from a group of 107 healthy normal subjects matched for age, gender and estimates of their basal (or pre-morbid for the patients) IQ, based on tests of irregular word reading (either the National Adult Reading Test – NART (Nelson & O’Connell, 1978), or the Wechsler Test of Adult Reading – WTAR (Wechsler, 2001; see Table 1)). In this control group, subjects were right-handed, native English speakers; they had no history of neurological or psychiatric disease, and were capable and willing to take part in the experiment. Patients were compared to controls using a voxel-based approach.

### 2.2 Experimental paradigm

#### 2.2.1 Prospective memory tasks and design (Fig. 2)

Participants were required to perform two types of prospective memory tasks: time-based and event-based, each of which used two kinds of material, words or pictures. The word and picture versions of each task were identical except for the stimuli presented. Each type of prospective memory task using each kind of material was performed in a separate session, yielding four different sessions. In the prospective memory tasks, participants were required to maintain an intention to act (the prospective memory task itself) while doing something else (called the ongoing task), and to retrieve and act upon this intention at the appropriate moment (see Burgess et al., 2001 for description of typical prospective memory designs).

In the ongoing tasks, two words (in the word version of the task) or two pictures (in the picture version) were shown on the display, side-by-side, in a horizontal orientation, in the middle of the screen. Participants were required to press the mouse key that was in the same direction (left/right) as the shortest word (in the word version) or the less heavy object (in the picture version). The order of left and right responses was pseudorandomised, with equal numbers of the two responses. All trials were self-paced with an upper limit of 3 s.

During each session, an ongoing phase was first performed, before any prospective memory instruction had been given. An initial practice phase of 10 trials long was given, aimed at orienting the participant to the ongoing task. Participants were not able to make mistakes (the program did not show the next stimulus until the correct response had been made). Then the ongoing task itself was given (‘OGonly trials’). Twenty ‘OGonly trials’ were analysed from this phase. After these two phases, came a third phase during which the prospective memory tasks themselves were introduced. During this phase (‘PM phase’) participants were asked to press a different key in certain situations while performing the ongoing task. The prospective memory instructions were not given before this phase. As participants also performed prospective memory tasks (‘PM trials’) in addition to ongoing trials during this phase, the latter are referred to as ‘OGPM trials’ during this phase. Both ongoing tasks and ‘PM trials’ were self-paced, with an upper limit on response times for all stimuli of 3 s. There were two types of ‘PM trials’, event-based and time-based.

In the *event-based prospective memory tasks*, subjects were instructed that if they saw an animal (either word or picture) they should press the spacebar instead of performing the ongoing task. Participants were encouraged to respond to the ‘PM target’ (i.e. press the spacebar) even if they were late in responding. In other words, participants performed the ongoing task, and when a specified situation occurred (animal = ‘PM target’) they had to engage prospective memory. Trials with ‘PM targets’ were defined as event-based ‘PM trials’. The number of ‘OGPM trials’ between each PM target was the same for all participants, and followed a fixed order. Specifically, the intervals were arranged as follows (30 24 22 16 14 8 6 2 4 10 12 18 20 26 28). The PM phase was composed of 242 OG trials, and 15 PM targets. The overall frequency of PM targets was 5.9% (the last two OG trials were not counted).

In the *time-based prospective memory task*, participants were asked to press the spacebar every 30 s while performing the ongoing task. In this condition, self-paced space bar presses were ‘PM trials’. Participants were given a stopwatch box to help them know the time. To inspect the stopwatch, they had to press a red button that opened the box and displayed the time that had elapsed since the start of the test phase. The program recorded the times at which the subject depressed, and then released the red button. The retention interval of 30 s for the time-based tasks was chosen, on the basis of pilot data, as a balance between five competing requirements. The first was that the retention intervals of the event- and time-based prospective memory tasks to be very broadly equivalent. The second requirement was to have accuracy performance in healthy controls close to ceiling, but not at ceiling. Experience has shown (e.g. Burgess & Shallice, 1996) that often the psychometric tests that best discriminate controls from different lesion groups within a neurological population do so because they measure best levels of impairment (rather than levels above the mean within the normal range). Third was the requirement to have a sufficient number of data points (i.e. responses) for statistical purposes. Fourth was the requirement for the task to be as short as possible, for practical and ethical reasons. The fifth reason for 30 s retention intervals was that this length was also broadly comparable to the kinds of retention intervals being adopted in neuroimaging studies, thus increasing the likelihood of potential cross-talk between this lesion study and neuroimaging ones.

The same principles of time- and event-based tasks (each including the three phases previously described) were used for both words and pictures in distinct sessions. Pictures were selected images of single items from the photographic collection “20,00 Photos” (Focus Multimedia Limited) including animals, cityscapes, landscapes, buildings, and vehicles, at a size of approximately 5 cm tall and wide. The images were picked to be of high frequency, easily nameable items, presented in cardinal views. The word stimuli were high frequency words between 3 and 12 letters long. One of the words in the pairs was always longer than the other, including on the PM trials (animal words).

The instructions for the time- and event-based prospective memory tasks were similar. For example, the instructions for the event-based task (using words) were: [Describing the ongoing task] “*In this task, you will be shown two words at a time like this: Nut Rucksack. And we would like you to press the mouse key in the direction of the shortest word. In this example, Nut is a shorter word than Rucksack, and is on the left-hand side of the screen, so you would press the left-hand mouse key.*” Participants were then given the practice session. After completion of the practice, they were told: “*That's the end of the practice. Now you will do it for real. We want you to press in the direction of the shortest word as fast as you can without making mistakes. If you are not quite sure, just have a guess, as you will only have a short time to think about it*”. Participants then undertook the ongoing-only phase. After this was complete, the instructions for the PM phase were given: “*Now we want you to do something extra. As well as pressing as fast as you can in the direction of the shortest word, this time if you notice that any of the words are ANIMAL words, then press the SPACEBAR on the keyboard instead. Sometimes when people are doing this task, they find that by the time they have noticed an animal word, they have already pressed a key on the mouse or the animal has already disappeared. If this happens to you, press the spacebar as soon as you can anyway. The most important thing is to register that you have noticed an animal word by pressing the spacebar, even if you are a bit late.*” The PM phase was then administered. For the time-based task (words) the instructions for the practice and ongoing-only phases were identical. For the PM phase, the instructions were: “*Now we want you to do something extra. As well as pressing as fast as you can in the direction of the shortest word, this time we would like you to press the SPACEBAR on the keyboard every 30 s while you are doing the test. To help you with the task, you can use a stopwatch. The experimenter will now show this to you.*” [The experimenter then demonstrated use of the stopwatch box and told the participants not to hold the red button which opened the box down but to release it after consulting the time.] “Remember that you have to press the spacebar every 30 s when doing the test, not just once.” The experimenter read through the instructions, which were presented on the computer display, with the participants, and the participants were required at each point to demonstrate understanding by pressing a particular key before proceeding. Any queries were answered by the experimenter as they occurred.

Performance of prospective memory tasks is a complex matter. There may be many contributing cognitive components which, while not regarded as central to the realisation of a delayed intention, nevertheless may contribute to performance on the tests. These include the ability to process and remember several instructions (i.e. those relating to both the ongoing task and also the PM targets); the ability to allocate attention between tasks, being able to monitor the environment for a cue, and then interrupt and inhibit ongoing activities. All of these abilities have been shown to be impaired, potentially, in frontal lobe patients. But what we are seeking in this study is to find a core prospective memory deficit which is independent of these kinds of secondary problems, as suggested by e.g. the work of Burgess and colleagues (e.g. Burgess et al., 2000; Shallice & Burgess, 1991). Therefore, in addition to the prospective memory tasks, we used a series of secondary tasks, administered separately, in order to control for potential problems in the following functions: (i) basic aspects of attention and speed (reaction times); (ii) response inhibition; (iii) problems remembering multiple instructions; (iv) task switching. We also used separate tasks in order to assess time estimation abilities in case this might be a cause of any time-based prospective memory difficulty, following the findings of Petrie (1952). As far as possible, the same stimuli were used in these additional tasks as were used in the prospective memory tasks, so as to minimise the irrelevant differences (i.e. those not important from an experimental viewpoint) between the tasks. Lists of the particular stimuli used are available from the authors.

#### 2.2.2 Basic attention tasks

These tasks were designed in order to control for basic aspects of attention, sensori-motor reaction times and vigilance. Two tasks were performed: Simple Reaction Time (SRT) and Preparatory Attending (PREP). In SRT, participants were shown pictures and words, and had to press the space bar as soon as the next item appeared. One hundred and twenty items were presented, with three possible pseudorandomly ordered inter-stimulus intervals: 0.5 s (40 times), 1 s (40 times) or 2 s (40 times). Stimulus duration was self paced.

PREP was similar as SRT, except that there were longer pauses between displays. 24 items were presented, with three possible inter-stimulus durations: 2.5 s (8 times), 5 s (8 times) or 10 s (8 times). This task therefore measured participants’ ability to maintain attending behaviour over an unfilled inter-stimulus interval, i.e. “preparatory attending”.

#### 2.2.3 Target detection and response inhibition (DETECT and INHIB)

These tasks were aimed at controlling for target detection (DETECT task) and response inhibition (INHIB task) difficulties. DETECT and INHIB were two complementary tasks. INHIB task was always performed after DETECT.

In DETECT, participants were shown a series of pictures, and had to the press space bar as soon as they saw an animal. The rest of the time they just had to watch the screen. 112 items were presented, including 10 targets (i.e. animals), minimum intervening trials between targets was 2 and maximum was 18, with number of intervening targets in fixed schedule: 10, 6, 12, 4, 18, 2, 16, 8, 14, 10. Stimulus duration was self paced with maximum of 1500 ms. Inter-trial intervals (blank screen) lasted 300 ms.

In INHIB, participants were shown a series of pictures and were asked NOT to press the spacebar when an animal is shown, but press the space bar when something else was shown. One hundred and twelve items were presented, including 10 targets (animals). Stimulus duration was self paced with maximum of 3000 ms. Target distribution was as for DETECT. Inter-trial intervals lasted 300 ms.

#### 2.2.4 Multiple instruction tasks (INSTRUCT, SWITCH)

These tasks required remembering and responding to several instructions during the same task. The first (INSTRUCT) required remembering a series of instructions. The second (SWITCH) was a standard task-switching paradigm. Each task used exactly the same categories of stimuli (words, numbers, pictures), with each stimuli type having a different operation that had to be performed with it. But the INSTRUCT and SWITCH tasks differed in the order that stimuli were presented. Stimuli of a particular type were presented in blocks in INSTRUCT, while they were randomly distributed in SWITCH.

In INSTRUCT participants were shown pairs of words, numbers or pictures. For words, they had to press mouse key in direction of word containing letter P. For pictures, they pressed in direction of cheapest item, for numbers they pressed in direction of the even number. Words, pictures and numbers were blocked. Ninety items were presented. Stimulus duration was self paced. Intertrial intervals lasted 300 ms.

SWITCH was composed of the same subtasks, except that words, numbers and pictures were not blocked but randomly intermingled. Thus INSTRUCT estimates the ability of a participant to remember multiple instructions, and SWITCH the ability to switch between them. These abilities are necessary components of a prospective memory task.

#### 2.2.5 Time estimation tasks (TE)

One of the demands that time-based prospective memory tasks often make, over and above those made by event-based prospective memory tasks, is the ability to maintain some estimate of the passage of time. This ability can be impaired in patients with frontal lobe damage (Petrie, 1952; Rubia, 2006). Accordingly, TE tasks were designed in order to evaluate the ability to estimate short and longer time intervals. Participants were first asked to count from 1 to 10 in time with a computer-displayed numeral in order to establish a pace, and then they were required, when the numerals ceased to be displayed, to count up to the other numbers (e.g. 11–20) at the same pace *in their head* (i.e. silently), and then press the space bar key when they reached the end. This task was composed of two parts.

In the first 4 trials (TE1), stimulus duration was 100 ms, and intertrial interval was 1000 ms. In other words, after the first 10 externally-paced numbers, participants had to estimate and produce 1 s time intervals by counting in their heads until reaching 20 (in the first trial), until 30 (in the second trial), 40 (in the third trial) and 50 (in the last trial). The global TE1 score summed the time estimated in each trial (ideal time: 10 + 20 + 30 + 40 = 100 s).

In the last 4 trials (TE2), stimulus duration was still 100 ms, but the inter-trial interval was 2000 ms. In other words, after the first 10 externally-paced numbers, participants had to estimate and produce 2 s time intervals, by counting in their heads until reaching 15 (in the first trial), 20 (in the second trial), 25 (in the third trial) and 30 (in the last trial). The global TE2 score summed the time estimated in each trial (ideal time: 10 + 20 + 30 + 40 = 100 s).

In order to facilitate direct comparison between these two sets of trials, in the fast pace trials (TE1) participants were asked to count to a higher number than in the slow pace trials (TE2). This allowed the total duration of the period over which they should have been estimating time duration to be identical (i.e. 100 s).

The whole experimental testing lasted approximately 1 h. The tasks were administered in the same fixed order in all participants: SRT; INSTRUCT; event-based prospective memory task (pictures); time-based prospective memory task (pictures); time-based prospective memory task (words); event-based prospective memory task (words); TE1; TE2; SWITCH; DETECT; INHIB; PREP.

### 2.3 Structural imaging

Patients underwent either a structural MRI (*n* = 35) or a CT scan (*n* = 10), in the context of their clinical or neuropsychological evaluation or follow up. Images were obtained at the neuroradiology departments of the collaborating hospitals, and were collected for clinical purposes only, in accordance with the ethics approvals. Accordingly, the scans were acquired using diverse acquisition sequences, depending on the machine and/or the patient's pathology. The MR images used for further processing were T2-weighted MRI as they were available for all the patients who underwent an MRI. T2-weighted scans, although offering less contrast precision than T1-weighted scans between grey and white matter, give good pathological information, by highlighting regions of damage. However, all available sequences were used by the neurologist (E.V.) in order to identify the limits of each lesion. Structural MR and CT images were converted into the SPM format (Statistical Parametric Mapping; http://www.fil.ion.ucl.ac.uk/spm/) for further processing described below.

### 2.4 Imaging and statistical analyses: voxelwise statistical method: AnaCOM

MRI images were pre-processed in SPM5 (http://www.fil.ion.ucl.ac.uk/; Wellcome Institute of Cognitive Neurology, London). The first step consisted in spatially normalizing MRIs to the Montreal Neurological Institute (MNI) template. As spatial normalization can be affected by the presence of a brain lesion, all signal abnormalities due to the lesion were first traced (using MRIcro, http://www.sph.sc.edu/comd/rorden/mricro.html) and were used as a mask during the normalization procedure to optimize the brain normalization. This ‘Cost Function Masking’ procedure (Brett, Leff, Rorden, & Ashburner, 2001; Crinion et al., 2007) was used to weight the normalization to brain rather than non-brain tissue or lesions. Both the ‘Cost Function Masking’ method and the more recent ‘Unified Model’ (Crinion et al., 2007) for normalizing brains were tested on our set of data. Visual inspection showed better results for the ‘Cost Function Masking’ procedure. This is in accordance with recent results (Andersen, Rapcsak, & Beeson, 2010), and may additionally be due to the fact that we used T2-weighted MRIs while grey and white matter differentiation (and thus segmentation) is greater on T1 images. The spatial normalized images were resliced with a final voxel size of 1 mm × 1 mm × 1 mm. The normalized images were then compared to the MNI template to evaluate normalization accuracy. The normalizing procedure failed for 6 patients. For these 6 patients, the segmentation followed the same procedure as for CT scans, as described below. For the remaining 29 successful normalizations, brain lesions were manually segmented again, this time on the normalized anatomical MRI, in order to extract the normalized lesion volume. This second segmentation was used for further statistical analyses.

CT images (and also MRIs which failed to normalize) were pre-processed differently because the SPM normalization was not possible. Normalization and segmentation were performed in one step, by directly reconstructing the lesion onto the MNI template. Patients’ lesions were drawn on the MNI template by a neurologist (E.V.) who was, at that time, blind to the scores of the patients. In order to facilitate the comparison of the patients’ space and the MNI space, and to improve the lesion transfer, the patients’ structural image was re-oriented to match the template orientation, in particular regarding the axial plan (pitch). This matching was performed using free rotations in the MRIcro software (http://www.sph.sc.edu/comd/rorden/mricro.html). To ensure the validity of combining CT and MRI scans, we run a separate analysis including only patients with an MRI, for each prospective memory task. The results were very similar to the ones obtained with all patients.

The analysis was performed using a recently developed voxel-by-voxel lesion mapping method, AnaCOM (see Kinkingnehun et al., 2007 for a full description of the method). AnaCOM permits statistical analysis of the voxels which explain the most variance in relation to a cognitive or behavioural deficit. The previously described normalization and segmentation steps resulted in a three-dimensional reconstruction of each patient's lesion. The next step consisted of weighting each of these lesion volumes by the score obtained by each patient in a given task. This was performed by attributing, to all the voxels of each lesion volume, the value of the score of the corresponding patient (for instance, if a patient scored 3/10 on a given task, all the voxels included in his brain lesion were set at 3), while the rest of the image was set to zero. Volumes representing each patient's lesion (*n* = 45) were then superimposed in order to built the “Maximum Overlap Map”. This map gave, for each voxel the number of lesions that include this voxel. In these maps, the patterns of overlaps of the segmented lesions defined subregions (group of voxels covered by the same lesions). Statistical analyses were performed in the subregions that were composed of at least three lesions. For these subregions, a non-parametric Wilcoxon rank-sum test was used, corrected for multiple comparisons (Bonferroni–Holm correction). This test compared performance of patients with damage to that subregion vs. control participants. Only regions where statistical significance at *p* < 0.05 was present after Holm correction were considered.

These steps were performed for the scores of each prospective memory task. Statistical maps were thus obtained for each score. Each statistical map represented brain regions where the patients’ performance statistically differed from that of the control subjects for a given task. These statistical maps thus indicated the clusters of voxels within the areas covered by at least three overlaps that contributed the most to a given impairment.

### 2.5 Behavioural statistical analyses

Statistical tests were performed using SPSS software (SPSS for windows, version 16, SPSS Inc., Chicago, IL). All demographic and behavioural data were tested for normality, and statistical tests were chosen consequently. When assumption of normality was met, *t* test or one-way ANOVAs and post hoc tests were used. Otherwise, Kruskal–Wallis tests and Mann–Whitney tests were chosen. We checked for any significant between-group differences in basic demographics, estimate of premorbid IQ (NART or WTAR), and lesion data (side, volume, aetiology). Then, we tested for between-group differences in prospective memory and control tasks.

## 3 Results

### 3.1 Patient data

No significant differences were found between patients and controls in terms of age at testing (*t* test: *t* = 0.96; *p* = 0.341; df = 150), gender (Pearson chi-square: *χ*^2^ (1) = 0.01; *p* = 0.753), or premorbid IQ estimated by the NART or WTAR (*t* test: *t* = 0.35; *p* = 0.727).

### 3.2 Behavioural data

Accuracy and RT on ‘OGonly trials’, ‘OGPM trials’, and ‘PM trials’ were analysed. Performance and statistical comparisons are summarized in Table 2 (time-based tasks) and Table 3 (event-based tasks).

Performance and statistics regarding control tasks, including Time Estimation (TE) are shown in Table 4.

### 3.3 AnaCOM results

#### 3.3.1 AnaCOM maps (Table 5; Fig. 3)

**Fig. 3:**
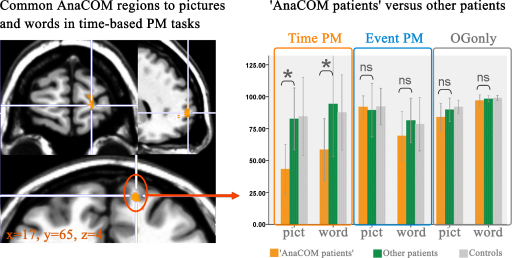
AnaCOM region common to picture and word time-based prospective memory. AnaCOM results are superimposed on axial, sagital and coronal views of a standard brain using MRIcron software. Damage in the region in orange was associated with a deficit in time-based prospective memory, (measured by the delay between two space bar presses) for both words and pictures, at a Holm threshold (*p* < 0.05 FWE corrected). Right side of the brain is on the right. Graphs show performances of patients damaged in that region ‘polar BA10 patients’, compared to performances of ‘other patients’, and of controls, in the different conditions. *Y*-axis shows performance expressed as a percentage of the ideal accuracy score on each condition (for the purpose of the graph showing performance in the different tasks, time-based ‘PM trials’ performance corresponds here to the real number of space bar presses, expressed as a percentage of the ideal number of space bar presses during the PM phase).

AnaCOM maps were built for ‘PM trials’ performance in each prospective memory task (event- and time-based for words and pictures). Locations and significance of deficits associated with the different prospective memory tasks are presented Table 5. Time-based ‘PM trials’ performance was associated with a right rostral prefrontal region (BA10), which was common for both picture and word versions of the task (Fig. 3). Event-based ‘PM trials’ performance was associated with distinct regions for words and pictures (see Table 5).

As AnaCOM compares patients to controls, we conducted an additional analysis in order to better examine the specificity of the prefrontal regions for time-based and event-based prospective memory tasks. For each AnaCOM region associated with a prospective memory deficit, we compared patients who were damaged in a given AnaCOM region to all the other patients whose lesion did not concern this region. For time-based tasks, patients damaged in the AnaCOM region within BA10 (‘polar BA10 patients’) were compared to all the other patients whose lesion did not concern this region (‘other patients’), and patients damaged in the AnaCOM region within BA47 (‘BA47 patients’) were compared to all the other patients whose lesion did not concern this region (‘other patients’). For event-based prospective memory, patients damaged in the AnaCOM region within BA32/10 (‘BA32/10 patients’) were compared to all the other patients whose lesion did not concern this region (‘other patients’) and patients damaged in the AnaCOM region in BA9 (‘BA9 patients’) were compared to all the other patients whose lesion did not concern this region (‘other patients’).

#### 3.3.2 ‘Polar BA10 patients’ vs. ‘other patients’ (Table 6; Fig. 3)

‘Polar BA10’ and ‘other patients’ did not differ in age at test (*U* = 97.5; *z* = −0.09; *p* = 0.930), in gender (Pearson *χ*^2^ (1) = 0.278; *p* = 0.598), in NART-FSIQ (difference between pre-morbid and post-morbid IQ; *U* = 13.5; *z* = −1.30; *p* = 0.200), in lesion volumes (*U* = 51.0; *z* = −1.77; *p* = 0.080), or lesion side (Pearson *χ*^2^ (2) = 5.43; *p* = 0.066).

We did not find any significant difference between ‘polar BA10’ and ‘other patients’ for the control tasks (excluding time estimation tasks that will be described below), for ‘OGonly’ (except for event-based tasks with words when looking at RT), and for event-based ‘PM trials’ (Fig. 3). ‘Polar BA10 patients’ differed significantly from ‘other patients’ only in time-based ‘PM trials’ (Table 6). Intervals between two space bar presses in time-based prospective memory tasks for pictures (‘PM trials’) were 48.084 ± 5.1 s (ideal interval: 30 s) for ‘polar BA10 patients’, 32.357 ± 8.7 s for ‘other patients’ and 32.934 ± 9.6 s for controls. For words, they were 47.802 ± 6.0 s for ‘polar BA10 patients’, 31.670 ± 10.2 s for ‘other patients’ and 30.147 ± 6.4 s for controls.

Time estimation for longer intervals (2 s rhythm – TE2) was significantly impaired in ‘polar BA10 patients’ compared to ‘other patients’ (TE2 score was 65.137 ± 21.8 s in ‘polar BA10 patients’ and 85.914 ± 29.5 s in ‘other patients’, the ideal score being 100 s; see Table 6 for statistics). Differences in TE1 scores were not significant (TE1 score was 81.562 ± 26.3 s in ‘polar BA10 patients’ and 94.870 ± 29.3 s in ‘other patients’).

Taken together, these results suggest that lesions in the right polar BA10 region are specifically associated with a deficit in time-based ‘PM trials’, but (a) are not associated with a deficit in event-based trials, (b) nor with a global deficit in ongoing task performance, and (c) this deficit could not be explained by deficits in basic attention, detection, inhibition or multiple instruction processing.

It is notable that ‘polar BA10 patients’ were slower than the ‘other patients’ in ‘OGPM trials’ during event-based tasks. However, as RT did not significantly increase from the ‘OGonly’ phases (before PM phases were encountered) to ‘OGPM’ trials (i.e. the ongoing trials during the PM phases), this slowness cannot be interpreted as a deficit relating to prospective memory (for picture event-based tasks, RT = 1739.2 ms in ‘OGonly’ and 1559.6 ms in ‘OGPM’ trials, *z* = −1.21; *p* = 0.225; for word event-based tasks, RT = 1505.1 ms in ‘OGonly’ and 1558.3 ms in ‘OGPM’ trials, *z* = −0.67; *p* = 0.500, Wilcoxon signed rank test). Incidentally, one might note that as the ongoing task was exactly the same in both the event- and time-based tasks, the fact that we found a difference in reaction time between ‘polar BA10’ and ‘other’ patients during the ‘OGonly’ phase of the experiment, only in event-based tasks for words, but not for the equivalent test using pictures, is curious, and defies interpretation at this time.

In addition to the prospective memory impairment, the ‘polar BA10 patients’ were significantly impaired in time estimation for 2 s intervals (TE2; see Table 6), compared to ‘other patients’, suggesting that these rostral patients may have time estimation problems when longer duration periods are involved, or when the current time estimation interval (i.e. 2 s in TE2) conflicts with a previous one (i.e. 1 s in TE1). However, correlations between ‘PM trials’ and TE2 performance were significant only for pictures in the whole patients group (all together: for pictures, *R*s = −0.344; *p* = 0.047; for words: *R*s = −0.020; *p* = 0.912), and not significant in controls (for pictures, *R*s = −0.107; *p* = 0.145; for words: *R*s = −0.046; *p* = 0.537).

In order to better evaluate the role of time estimation in our tasks, we looked at the number of times participants checked the clock in time-based tasks. As a clock was available for the participants in these tasks, the requirement for time estimation was reduced. However, it is possible that good time estimation abilities might nevertheless make the task easier, or that some participants chose to estimate time rather than to use the clock. Number of clock checks was available for only three rostral patients, and the difference between ‘polar BA10 patients’ and ‘other patients’ did not reach significance (*U* = 16.5; *z* = −1.56; *p* = 0.119; mean number of clock checks among ‘polar BA10 patients’ across tasks = 7.3 ± 4.9; among ‘other patients’ = 15.0 ± 7.7; and among controls mean number of clock checks was 16.3 ± 8.9). However, in both all patients and controls, there was a significant correlation between time-based ‘PM trials’ performance (average delay between two space bar presses) and mean number of clock checks, for both pictures (patients: Spearman rho *R*s = −0.539; *p* = 0.007; controls: *R*s = −0.400; *p* = 0.004) and words (patients: *R*s = −0.671; *p* < 0.001; controls: *R*s = −0.459; *p* = 0.001). In other words, patients and controls with poorer time-based prospective memory performance checked the clock less frequently.

#### 3.3.3 ‘BA47 patients’ vs. ‘other patients’ (Table 7)

Patients who were damaged in the AnaCOM region in right area 47 were impaired (as expected from the whole brain analysis) in time-based task for pictures, but they were also impaired in time-based tasks for words, suggesting that this region is not specific for pictures. By contrast, these patients were not impaired significantly compared to other patients in event-based tasks. They had no deficit in ongoing tasks and in our set of control tasks, suggesting that the AnaCOM region in BA47 plays a critical role in time-based prospective memory.

#### 3.3.4 ‘BA9 patients’ vs. ‘other patients’ (Table 8)

Patients who were damaged in the AnaCOM region in left area 9 were impaired (as expected from the whole brain analysis) in event-based task for pictures, but they were not impaired in any other event- or time-based tasks. These patients had no deficit in ongoing tasks and in our set of control tasks, suggesting that the AnaCOM region in left BA9 plays a critical role in event-based prospective memory for pictures.

#### 3.3.5 ‘BA32/10 patients’ vs. ‘other patients’ (Table 9)

Patients who were damaged in the AnaCOM region in anterior cingulate area (BA32/10) were not specifically impaired in prospective memory tasks, but rather show non specific deficits in almost all ongoing or control tasks. More precisely, these deficits concerned almost exclusively slowness in RT. This suggests that prospective memory impairment in patients with damage to BA32/10 is secondary to other deficits.

## 4 Discussion

We studied 45 patients with diverse focal brain lesion, and compared them to 107 controls matched for age, gender and premorbid IQ. We administered time-based and event-based prospective memory tasks, using two types of material, words and pictures. In parallel we used several additional tasks in order to control for basic attention and reaction times, inhibition and processing of multiple instructions. We then examined brain-behaviour relationships with the voxelwise AnaCOM method. We found a polar right BA10 region (‘polar BA10’) and an inferolateral prefrontal area (‘BA47’) associated with a specific deficit in time-based ‘PM trials’ for both words and pictures. Compared to all the other patients, patients with lesions in this ‘polar BA10’ region, or patients with lesion in ‘BA47 region’, were significantly impaired in time-based ‘PM trials’ for both pictures and words, but they were not impaired in our various control tasks. Nor were they globally impaired on the ongoing tasks, and they were also not impaired in event-based prospective memory performance. Event-based prospective memory deficits were instead associated with damage to other regions, notably the left posterior dorsolateral prefrontal cortex (BA9) that was specific for pictures, and the medial prefrontal/anterior cingulate cortex (BA32/10).

This is the first lesion study showing crucial regions for prospective memory. Very few previous lesion studies have explored time-based and event-based prospective memory (Brooks, Rose, Potter, Jayawardena, & Morling, 2004; Cheng, Wang, Xi, Niu, & Fu, 2008; Cockburn, 1996). These have yielded inconsistent results regarding the existence of a deficit in event- or time-based prospective memory after brain damage. Further, these studies used a global approach at a brain or a lobar level, comparing all pooled patients to controls, and thus did not allow conclusions to be drawn about specific prefrontal subregions.

In contrast, the current study points to specific areas, and suggests in addition that time-based and event-based prospective memory might be supported at least in part by distinct regions. In functional MRI or PET, most of the published studies have examined event-based rather than time-based prospective memory (Burgess et al., 2001, 2003; Gilbert et al., 2009; Okuda et al., 1998, 2007; den Ouden et al., 2005; Reynolds et al., 2009; Simons et al., 2006). These functional imaging works highlighted the involvement of rostral PFC in event-based tasks. Our lesion results did not reproduce these event-based findings, but did suggest the involvement of other regions (posterior lateral PFC ‘BA9 region’ and anterior cingulate cortex ‘BA32/10 region’), that were frequently activated in these studies (Burgess et al., 2001; Gilbert et al., 2009; Okuda et al., 1998; den Ouden et al., 2005; Reynolds et al., 2009; Simons et al., 2006). Further analyses focused on AnaCOM regions showed that prospective memory deficit in patients damaged in ‘BA32/10 region’ may be explained by a non specific slowness of responses, observed in almost any task, including simple RT task. This result may relate to the role of medial prefrontal and adjacent cingulate cortex in motor initiation or energization (Shallice, Stuss, Alexander, Picton, & Derkzen, 2008; Stuss & Alexander, 2007; Stuss et al., 2005). Conversely, left ‘BA9 region’ appeared specific to event-based tasks for pictures. Dorsolateral prefrontal regions have been shown activated in functional imaging studies, though without a clear lateralization (Okuda et al., 1998; Reynolds et al., 2009; Simons et al., 2006). A recent TMS study showed the importance of region dorsolateral prefrontal region for event-based prospective memory (Basso, Ferrari, & Palladino, 2010).

**Fig. 4:**
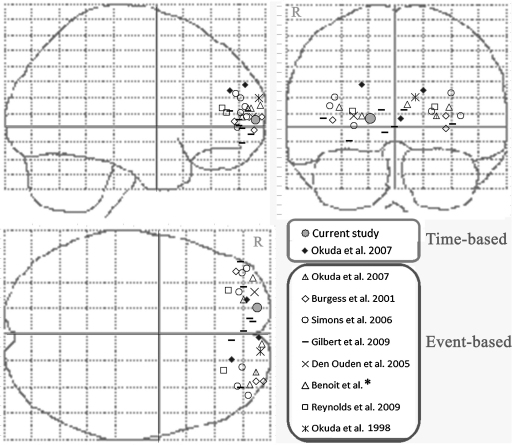
Comparative MNI coordinates of the current study and previous published neuroimaging results concerning rostral prefrontal activation. Maxima of activation reported in these functional imaging studies and in the current study are projected on a glass brain in the MNI space (*: in preparation). For each study, all maxima falling within BA10 for each contrast using prospective memory tasks are reported, excluding deactivation. R: right.

Time-based prospective memory was examined in one recent fMRI study (Okuda et al., 2007) that showed, in convergence with the current results, a polar (and medial) rostral PFC activation, located close to the AnaCOM ‘BA10 region’ we found in relation specifically to time-based tasks (see Fig. 4). This time-based specific region was involved using both words and pictures, suggesting that right polar rostral BA10 is not dependent of the type of stimulus processed in prospective memory, as also observed in other previous functional imaging studies using conjunction designs with spatial, verbal, visual, numerical materials (Burgess et al., 2001, 2003; Gilbert, Frith, & Burgess, 2005; Ramnani & Owen, 2004). The involvement of BA47 was not reported in the previous fMRI study when exploring time-based prospective memory (Okuda et al., 2007), and was observed in some event-based studies, in particular in relation to intention retrieval (Gilbert et al., 2009; Simons et al., 2006).

The critical role of polar rostral PFC for time-based prospective memory could be explained by at least two complementary hypotheses: a time estimation hypothesis (Graf & Grondin, 2006) and an intention retrieval hypothesis (Harris, 1982; Sellen, Louie, Harris, & Wilkins, 1997). Here, ‘BA10 patients’ were impaired in time estimation task for longer intervals, in addition to their impairment in time-based prospective memory. That rostral PFC may be involved in the subjective passage of time dates (as far as we are aware) back to Petrie (1952). In functional imaging, the rostral PFC has not been consistently found to be activated by time estimation tasks (Pouthas et al., 2005; Rubia, 2006; Rubia & Smith, 2004; Wiener, Turkeltaub, & Coslett, 2010). Previous lesion studies suggest a role for prefrontal regions in timing abilities (Coslett, Shenton, Dyer, & Wiener, 2009; Koch, Oliveri, Carlesimo, & Caltagirone, 2002; Wiener & Coslett, 2008), especially on the right hemisphere (Harrington, Haaland, & Knight, 1998), but they did not specifically explore the involvement of the rostral prefrontal region in time estimation or production. A recent lesion study has nevertheless emphasised a role for this region in prospective time estimation (Picton, Stuss, Shallice, Alexander, & Gillingham, 2006) and there is also recent concordant evidence from a functional MRI study (Morillon, Kell, & Giraud, 2009). Picton and collaborators’ lesion study (Picton et al., 2006) used a motor timing task with a tone-paced and a self-paced condition, and pointed to a right rostral prefrontal region within BA10, in which damage was associated with deterioration in timing performance over time. However, this region seemed more dorsal than the region we observed in the current study. Morillon et al. (2009) used a perceptive duration estimation task varying both absolute and relative durations of events. A medial network including BA10 (MNI maxima: −18, 62, 24 and −6, 54, 2) was activated with time estimation tasks. These regions were involved in automatic time tracking for long intervals, and their activation was positively correlated with stimulus duration (the authors used a range of intervals from 100 ms to 8400 ms). In the present study, ‘polar BA10 patients’ underestimated time duration, as they pressed space bar with longer intervals than expected. This underestimation of time in right rostral frontal patients might explain their poor time-based prospective memory performances with normal event-based prospective memory abilities (Block & Zakay, 2006). However, we cannot affirm for sure that time misestimation is the reason for prospective memory problems in our patients. Indeed, in our study, participants could check a clock whenever they wanted to, which considerably reduced time estimation requirements. In other words, a participant could still perform our time-based prospective memory tasks even if he/she was not able to estimate time correctly, using the external clock.

A second plausible interpretation of our results is that rostral prefrontal patients may fail the time-based ‘PM trials’ because they fail to retrieve their intention to act (i.e. to remember to press space bar every 30 s). This interpretation is supported by several functional MRI studies, showing rostral activation in relation to the maintenance or retrieval of an intention, both in prospective memory tasks (Simons et al., 2006), or outside the frame of prospective memory (Bengtsson, Haynes, Sakai, Buckley, & Passingham, 2009; Forstmann, Brass, Koch, & von Cramon, 2005; Haynes et al., 2007; Poppenk, Moscovitch, McIntosh, Ozcelik, & Craik, 2010; Sakai & Passingham, 2003). It is likely that intention retrieval may involve different cognitive processes in time- than in event-based prospective memory, because in time-based tasks there is no external cue that indicates the appropriate moment for retrieving the intention (McDaniel & Einstein, 2007). Time-based tasks may require a greater frequency of intention thoughts than event-based ones (Sellen et al., 1997). By contrast, in event-based, external cues (an animal on the screen) can trigger the recall of the intention (Block & Zakay, 2006; Einstein, McDaniel, Richardson, Guynn, & Cunfer, 1995; Sellen et al., 1997). Thus, in time-based tasks, the intention has to be internally or spontaneously retrieved, potentially placing greater demands on self-initiated processing. The present results can therefore be linked with accounts of rostral PFC function emphasising its role in self-initiated, internally generated behaviour (Burgess, Gilbert, & Dumontheil, 2007; Christoff, Ream, Geddes, & Gabrieli, 2003). It could be argued that our time-based task is not a “pure” time based task (Graf & Grondin, 2006) because there is an external clock which can serve as an external cue for retrieving the intention. Thus this task may have both event and time components. However, it is still placing greater demands on self-initiated processing since participants could use it as many times as they wanted to and have to decide by themselves when to use it by clicking on the button (as opposed to external cues, which the participant cannot control).

Despite its importance in everyday life, the nature of retrieval in time-based prospective memory, i.e. how our intentions come to mind at the right time with no external cues, is a crucial and unresolved question. Several hypotheses have been proposed regarding intention retrieval in time-based prospective memory (Harris, 1982; Kvavilashvili & Fisher, 2007; Sellen et al., 1997): a spontaneous and periodic pop-up of the forthcoming task into one's mind, incidental cuing by environmental triggers or by internal thoughts, that remind about the intended task, and self-initiated effortful monitoring of planning thoughts. Recently, Kvavilashvili and Fisher (2007) tested these hypotheses in a naturalistic study in healthy subjects, by analysing in which circumstances intention retrievals occurred. The authors showed that all types of rehearsal occurred in a naturalistic task, with a predominance of those triggered by something incidental (67%), a minority of self-initiated planning thoughts (9%) and 24% of spontaneous pop-ups of the intention. There was a correlation between the number of retrievals and performance on the prospective memory task. It is not possible to conclude in our study which of these mechanisms was impaired in rostral patients. Nevertheless, we can assess indirectly the number of intention retrievals by the number of clock checks, as participants were likely to check the clock when they remembered that something has to be done soon (Ceci & Bronfenbrenner, 1985; Harris, 1982; McDaniel & Einstein, 2007; McFarland & Glisky, 2009). The number of clock checks was correlated with time-based performance in both patients and controls, suggesting that our participants relied on the clock to perform time-based tasks. This might suggest that rostral patients forgot to retrieve that they had something to do in a few seconds, in other words, their intention to act.

The preserved performances of frontopolar patients on event-based tasks may suggest that they remained sensitive to external cues and succeeded in retrieving the intention when externally triggered. Alternatively, one can suppose that one intact hemisphere may be sufficient to perform our event-based prospective memory tasks. Indeed functional imaging shows both left and right prefrontal regions co-activated in relation to event-based prospective memory tasks. However, as the number of rostral patients is low (*n* = 8), and right and left lesions were not homogenously distributed within the frontal lobes, the absence of an association between rostral PFC and event-based performance may reflect lack of power. Consistent with this possibility, Uretzky and Gilboa (2010) have recently described a frontopolar patient who presented with impaired event-based prospective memory and cue detection. Additionally, our included lesions did not cover all brain regions, precluding inferences about negative results.

More broadly, our results are concordant with previous lesion studies using specific multitasking assessments (Burgess et al., 2000; Dreher, Koechlin, Tierney, & Grafman, 2008). Burgess et al. (2000) used the Greenwich test, a multitasking test which requires the ability to follow arbitrary rules while engaged in a series of ongoing activities. Thus the performance reflects to a large degree “activity-based” prospective memory abilities. They showed a left rostral prefrontal region associated with impaired performance on this task. But when using another multitasking test, the Six Element Test, where performance reflects time-based self-paced task switching, these authors found the right rostral PFC to be crucial for multitasking (Burgess et al., 2008). Taken together, these studies may suggest the possibility of specialized roles for left and right rostral regions, with the relative contributions of each differing according to whether intentions are time- or activity-based. Given the involvement of rostral PFC regardless of the material used in prospective memory tasks both in functional imaging (Burgess et al., 2001, 2003; Gilbert et al., 2005; Ramnani & Owen, 2004) and in the current study, it is unlikely that this left/right specialization is dependent on the domain of information (i.e. verbal or spatial).

In more posterior regions, the lateral prefrontal region associated with time-based deficit was right-lateralized while the one associated with event-based deficit was on the left side. Previous lesion studies have suggested a lateralization of function in lateral PFC, the left lateral PFC being related to ‘task setting’ and the right lateral PFC to ‘monitoring and checking the task over time’ (Stuss & Alexander, 2007). Theoretically, event-based tasks may place more demand on ‘task setting’ (“if there is an animal, I press the space bar”), while time-based tasks may require more ‘monitoring’ of time information. But again, since right and left lesions were not homogeneously distributed within the frontal lobes, we are unable to be sure if controlateral homologues to the regions identified in the present study are also crucial for prospective memory.

In sum, the present findings showed that a polar part of right BA10 is crucial for time-based prospective memory, plausibly due to its role in estimating long time durations and/or in the self-retrieval of one's intention to act. These results are consistent with the view that the deficit of frontopolar patients in multitasking situations, i.e. their “strategy application disorder”, could at least in part be explained by a deficit in prospective memory (Burgess et al., 2008). Consistent with the present result, a meta-analysis by Gilbert et al. (2006) demonstrated that studies using multiple tasks tended to activate the polar part of BA10, while mentalizing and episodic retrieval were associated with more posterior BA10 subregions. Prospective memory is a new and useful window to better understand rostral patients’ problems and to explore the cognitive processes that depend on rostral prefrontal cortex. Nevertheless, it is likely that prospective memory is not the only crucial component that could explain the difficulties of patients with frontopolar damage.

## Figures and Tables

**Fig. 2:**
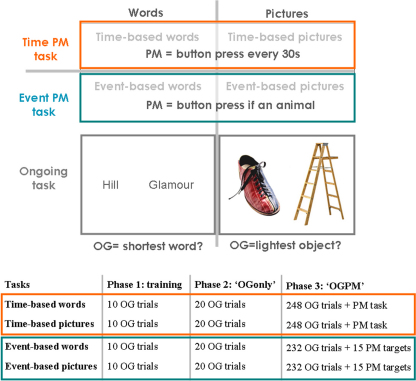
Experimental prospective memory tasks and design. In phase 2, there were 30 ‘OGonly trials’ in each task, among which the first 10 trials were discarded from analysis, leaving 20 analysed ‘OGonly trials’. OG: ongoing; PM: prospective memory.

**Table 1:** Characteristics of the patients included in the studies, grouped by lesion location. Among patients suffering for tumours, 18 presented with a glial tumour, 6 with a meningioma and 4 with another or unknown aetiology. Vascular patients had either ischemia (*n* = 3) or haemorrhage (*n* = 10) due to the rupture of a vascular malformation. PF: prefrontal; SD: standard deviation.

	All patients *n* = 45	Rostral PF *n* = 8	Posterior PF *n* = 17	Non PF *n* = 20	Controls *n* = 107
Mean (SD) Min–Max					
Age (years)	47.6 (10.8) 26–67	48.1 (12.5) 26–62	48.7 (10.9) 26–67	47.1 (10.1) 27–64	49.9 (14.5) 17–81
NART or WTAR (premorbid IQ)	103.5 (13.8) 74–124	100.7 (19.2) 76–120	103.9 (16.2) 74–124	104.1 (10.4) 90–124	104.3 (11.3) 77–126
WAIS-FSIQ	94.5 (14.9) 67–124	82.5 (16.4) 67–113	96.2 (14.3) 74–124	97.2 (13.7) 70–119	
Lesion volume (cm^3^)	49.8 (82.9) 0.8–464.9	101.3 (148.5) 10.9–465	46.6 (77.5) 2.5–332.8	33.5 (36.2) 0.8–95.4	
Time interval^a^ (months)	8.6 (11.4) 1–69	5.1 (6.2) 1–19	13.6 (16.3) 1–69	5.6 (4.3) 1–19	

Gender					
Male	23	4	10	9	51%
Female	22	4	7	11	49%
Lesion side					
Right	19	4	9	6	
Left	22	2	7	13	
Bilateral	4	2	1	1	
Lesion type					
Vascular	13	2	3	8	
Tumoural	28	5	13	10	
Other	4	1	0	3	

a Time interval: period of time separating the neuropsychological evaluation and the brain imaging

**Table 2:** Performances (mean (SD) Min–Max) in time-based prospective memory tasks. Times are generally in milliseconds and accuracy in percentage of correct answers. ‘Average time between two space bar presses’ is a measure of performance in time-based ‘PM trials’, and is given in seconds (ideal time between two presses: 30 s). Significant differences between patients and controls appear in bold font (performance accuracy in time-based PM trials refers to average delay between two space bar presses). RT: reaction times; PM: prospective memory; OGonly: performance in ongoing task trials in the phase where they performed alone (before a prospective memory instruction has been given); OGPM: performance in ongoing task trials in the phase where they are mixed with PM task; PM trials: only performance in prospective memory trials is considered.

	All patients *n* = 45	Controls *n* = 107	Patients vs. controls (Mann–Whitney tests)
**Time-based PM pictures**			
Accuracy in ‘OGonly’	89.2 (9.3) 65–100	92.0 (5.4) 80–100	*U* = 2038; *z* = −1.04; *p* = 0.300
RT in ‘OGonly’	1306 (339) 809–2267	1067 (197) 590–1640	***U***** = 1334;*****z***** = −3.96;*****p***** < 0.001**
Accuracy in ‘OGPM’	85.6 (7.8) 62.1–96.0	87.6 (3.7) 74.4–94.8	*U* = 2125; *z* = −0.65; *p* = 0.518
RT in ‘OGPM’	1163 (286) 803–2072	987 (167) 605–1351	***U***** = 1580;*****z***** = −2.93;*****p***** = 0.003**
Average time between two space bar presses	34.207 (9.796) 16.979–52.582	32.935 (9.579) 10.655–72.386	*U* = 1400; *z* = −0.36; *p* = 0.718
**Time-based PM words**			
Accuracy in ‘OGonly’	98.0 (4.0) 80–100	98.9 (2.7) 80–100	*U* = 2035; *z* = −1.32; *p* = 0.188
RT in ‘OGonly’	817 (320) 522–2102	635 (130) 385–973	***U***** = 1390;*****z***** = −3.66;*****p***** < 0.001**
Accuracy in ‘OGPM’	89.9 (8.5) 50.8–98.8	91.8 (3.5) 79.4–97.2	*U* = 2009; *z* = −1.05; *p* = 0.293
RT in ‘OGPM’	930 (280.6) 596–1983	774 (158) 450–1278	***U***** = 1452;*****z***** = −3.40;*****p***** = 0.001**
Average time between two space bar presses	33.625 (11.108) 11.394–70.175	30.147 (6.384) 11.336–56.837	*U* = 1156; *z* = −1.39; *p* = 0.164

**Table 3:** Performances (mean (SD) Min–Max) in event-based prospective memory tasks. Times are in milliseconds and accuracy in percentage of correct answers. Significant differences between patients and controls appear in bold font. RT: reaction times; PM: prospective memory; ‘OGonly’: performance in ongoing task trials in the phase where they performed alone (before a prospective memory instruction has been given); ‘OGPM’: performance in ongoing task trials in the phase where they are mixed with PM task; ‘PM trials’: only performance in prospective memory trials is considered.

	All patients *n* = 45	Controls *n* = 107	Patients vs. controls (Mann–Whitney tests)
**Event-based PM pictures**			
Accuracy in ‘OGonly’	90.3 (10.6) 40–100	94.3 (5.2) 80–100	***U***** = 1824;*****z***** = −2.26;*****p***** = 0.024**
RT in ‘OGonly’	1248 (364) 708–2659	1131 (230) 632–1794	***U***** = 1476;*****z***** = −3.60;*****p***** < 0.001**
Accuracy in ‘OGPM’	91.7 (11.9) 40–99.6	95.8 (2.6) 87.1–99.6	***U***** = 1871;*****z***** = −1.98;*****p***** = 0.047**
RT in ‘OGPM’	1271 (330) 816–2103	1066 (200) 618–1592	***U***** = 1479;*****z***** = −3.58;*****p***** < 0.001**
Accuracy in ‘PM trials’	83.5 (30.3) 0–100	93.0 (13.2) 13.3–100	*U* = 2135; *z* = −0.79; *p* = 0.432
RT in ‘PM trials’	1085 (416) 604–2177	879 (257) 499–1644	***U***** = 1394;*****z***** = −3.30;*****p***** = 0.001**
**Event-based PM words**			
Accuracy in ‘OGonly’	95.4 (9.6) 60–100	97.3 (4.3) 80–100	*U* = 1949; *z* = −0.11; *p* = 0.910
RT in ‘OGonly’	1037 (399) 598–2279	816 (177) 489–1251	***U***** = 1302;*****z***** = −3.10;*****p***** = 0.002**
Accuracy in ‘OGPM’	92.3 (12.7) 42.7–99.6	95.9 (6.9) 49.6–99.6	***U***** = 1530;*****z***** = −2.05;*****p***** = 0.041**
RT in ‘OGPM’	1200 (348) 709–2277	974 (215) 516–1832	***U***** = 1177;*****z***** = −3.68;*****p***** < 0.000**
Accuracy in ‘PM trials’	76.6 (24.4) 0–100	78.3 (21.5) 0–100	*U* = 1941; *z* = −0.13; *p* = 0.893
RT in ‘PM trials’	1288 (375) 706–2316	1102 (336) 649–2331	***U***** = 1234;*****z***** = −2.92;*****p***** = 0.003**

**Table 4:** Performances in control tasks for patients and controls. Times are in milliseconds (except for TE tasks) and accuracy in percentage of correct answers. Significant differences between patients and controls appear in bold font.

	All patients *n* = 45	Controls *n* = 107	Patient vs. control (Mann–Whitney tests)
Simple reaction time (RT)	501 (369) 243–2039	351 (85) 235–873	***U***** = 1545;*****z***** = −3.64;*****p***** < 0.001**
Preparatory attending (RT)	523.4 (289.8) 266.3–684.1	407.8 (80.6) 266.3–1983.5	***U***** = 1547;*****z***** = −3.28;*****p***** = 0.001**
Detection task (RT)	619 (111) 443–989	562 (111) 400–562	***U***** = 1569;*****z***** = −3.39;*****p***** = 0.001**
Detection task (errors)	0.1 (0.3) 0–1	0.1 (0.3) 0–1	*U* = 2289; *z* = 0.93; *p* = 0.355
Inhibition task (RT)	524 (156) 309–1079	470 (93) 313–890	*U* = 1969; *z* = −1.77; *p* = 0.077
Inhibition task (errors)	1.2 (1.3) 0–6	0.97 (1.33) 0–10	*U* = 2130; *z* = −1.19; *p* = 0.233
Multiple instruction task (RT)	1094 (328) 660–1990	914 (171) 606–1366	***U***** = 1630;*****z***** = −3.14;*****p***** = 0.002**
Multiple instruction task (errors)	2.1 (2.6) 0–12	2.2 (3.6) 0–21	*U* = 2297; *z* = −0.46; *p* = 0.646
Switching task (RT)	1115 (386) 696–2077	897 (166) 571–1535	***U***** = 1556;*****z***** = −3.37;*****p***** = 0.001**
Switching task (errors)	0.7 (1.7) 0–9	0.5 (0.9) 0–5	*U* = 2344; *z* = −0.20; *p* = 0.841
Time estimation 1 (short)	93.4 (29.0)	96.2 (17.1)	*U* = 2066; *z* = −1.30; *p* = 0.194
In seconds	2.8–221.2	22.0–126.1	
Time estimation 2 (long)	83.6 (29.3)	89.1 (22.9)	*U* = 1909; *z* = −1.93; *p* = 0.053
In seconds	13.4–209.1	2.9–172.4	

**Table 5:** Anatomical regions identified by AnaCOM maps to be significantly associated with a deficit in the different PM tasks. All the reported regions were significant after Holm correction for multiple comparisons (*H*: Holm threshold for significance). (BA = Brodmann area; G. = gyrus). For time-based task, the variables were the delay between two presses. For event-based tasks, they were mean reaction times.

PM tasks	Anatomical regions	BA	MNI coordinates			*p*-Values (×10^−3^)
Time-based PM pictures, *H* < 3.21 × 10^−3^	Right medial sup. frontal and orbital G.	10	13	51	−8	2.33
	Right superior frontal G.	10	17	65	4	3.21
	Right anterior cingulate G.	32/10	13	43	5	3.21
	Right inferior frontal G.	47	28	29	5	3.21
Time-based PM words, *H* < 1.30 × 10^−3^	Right superior frontal G.	10	17	65	4	1.04
Event-based PM pictures, *H* < 1.04 × 10^−2^	Left superior/middle frontal G.	9	−24	38	44	9.88
Event-based PM words, *H* < 1.07 × 10^−2^	Right anterior cingulate G.	32/10	14	45	3	10.7

**Table 6:** ‘Polar BA10 patients’ compared to all ‘other patients’. Significant differences appear in bold font.

	OGonly	OGPM	PM
Time-based pictures			
Accuracy	*U* = 70.0; *z* = −0.96; *p* = 0.364	*U* = 66.0; *z* = −1.10; *p* = 0.271	***U***** = 8.0;*****z***** = −2.78;*****p*** = 0.005*
RT	*U* = 59.0; *z* = −1.36; *p* = 0.185	*U* = 48.0; *z* = −1.78; *p* = 0.075	
Time-based words			
Accuracy	*U* = 79.5; *z* = −0.75; *p* = 0.568	*U* = 88.0; *z* = −0.27; *p* = 0.791	***U***** = 8.0;*****z***** = −2.76;*****p*** = 0.006*
RT	*U* = 48.0; *z* = −1.78; *p* = 0.078	*U* = 54.0; *z* = −1.55; *p* = 0.120	
Event-based pictures			
Accuracy	*U* = 91.0; *z* = −2.25; *p* = 0.830	*U* = 89.5; *z* = −2.30; *p* = 0.767	*U* = 68.5; *z* = −1.16; *p* = 0.246
RT	*U* = 54.0; *z* = −1.61; *p* = 0.114	***U*** **=** **43.0;*****z*** **=** **−2.02;*****p*** **=** **0.044**	*U* = 64.0; *z* = −1.04; *p* = 0.300
Event-based words			
Accuracy	*U* = 72.5; *z* = −0.66; *p* = 0.610	*U* = 44.5; *z* = −1.71; *p* = 0.088	*U* = 69.0; *z* = −0.68; *p* = 0.497
RT	***U***** = 28.0;*****z***** = −2.39;*****p***** = 0.014**	***U***** = 38.0;*****z***** = −1.97;*****p***** = 0.048**	*U* = 69.0; *z* = −0.49; *p* = 0.625

Control tasks	Errors	RT
Simple RT		*U* = 59.0; *z* = −1.53; *p* = 0.125
Preparatory attending		*U* = 61.0; *z* = −1.41; *p* = 0.159
Target detection	*U* = 92.5; *z* = −0.46; *p* = 0.646	*U* = 61.5; *z* = −1.39; *p* = 0.164
Response inhibition	*U* = 94.0; *z* = −0.23; *p* = 0.820	*U* = 54.0; *z* = −1.66; *p* = 0.097
Multiple instruction	*U* = 79.5; *z* = −0.76; *p* = 0.448	*U* = 58.0; *z* = −1.52; *p* = 0.129
Task switching	*U* = 88.5; *z* = −0.53; *p* = 0.595	*U* = 59.0; *z* = −1.48; *p* = 0.139
Time estimation	TE1 (short): *U* = 81.0; *z* = −0.69; *p* = 0.493	TE2 (long): ***U***** = 44.0;*****z***** = −2.02;*****p***** = 0.043**

RT: reaction times; PM: prospective memory; OGonly: performance in ongoing task trials in the phase where they performed alone (before a prospective memory instruction has been given); OGPM: performance in ongoing task trials in the phase where they are mixed with PM task; PM trials: only performance in prospective memory trials is considered.

* Performance accuracy in time-based PM trials refers to the average delay, calculated for each participant, between two space bar presses).

**Table 7:** ‘BA47 patients’ (from AnaCOM map of time-based tasks for pictures) compared to all ‘other patients’. Significant differences are highlighted.

	OGonly	OGPM	PM
Time-based pictures			
Accuracy	*U* = 43.0; *z* = −1.44; *p* = 0.150	*U* = 73.5; *z* = −0.11; *p* = 0.915	***U***** = 11.0;*****z***** = −2.59;*****p*** = 0.010*
RT	*U* = 74.0; *z* = −0.09; *p* = 0.932	*U* = 67.0; *z* = −0.39; *p* = 0.700	
Time-based words			
Accuracy	*U* = 31.0; *z* = −1.74; *p* = 0.081	*U* = 56.0; *z* = −0.12; *p* = 0.903	***U***** = 4.0;*****z***** = −2.55;*****p*** = 0.011*
RT	*U* = 43.0; *z* = −0.76; *p* = 0.449	*U* = 49.0; *z* = −0.46; *p* = 0.643	
Event-based pictures			
Accuracy	*U* = 59.5; *z* = −0.80; *p* = 0.423	***U*** **=** **28.0;*****z*** **=** **−2.09;*****p*** **=** **0.036**	*U* = 77.5; *z* = −0.02; *p* = 0.982
RT	*U* = 56.0; *z* = −0.92; *p* = 0.358	*U* = 67.0; *z* = −0.46; *p* = 0.646	*U* = 62.0; *z* = −0.45; *p* = 0.652
Event-based words			
Accuracy	*U* = 53.5; *z* = −0.86; *p* = 0.389	*U* = 48.0; *z* = −0.95; *p* = 0.340	*U* = 67.0; *z* = −0.05; *p* = 0.962
RT	*U* = 58.0; *z* = −0.48; *p* = 0.634	*U* = 63.0; *z* = −0.24; *p* = 0.812	*U* = 43.0; *z* = −1.06; *p* = 0.290

Control tasks	Errors	RT
Simple RT		*U* = 37.0; *z* = −1.83; *p* = 0.067
Preparatory attending		*U* = 64.0; *z* = −0.72; *p* = 0.473
Target detection	*U* = 71.5; *z* = −0.71; *p* = 0.477	*U* = 64.0; *z* = −0.72; *p* = 0.473
Response inhibition	*U* = 71.0; *z* = −0.46; *p* = 0.645	*U* = 68.0; *z* = −0.56; *p* = 0.577
Multiple instruction	*U* = 57.5; *z* = −1.00; *p* = 0.316	*U* = 57.5; *z* = −1.00; *p* = 0.577
Task switching	*U* = 70.0; *z* = −0.59; *p* = 0.557	*U* = 67.0; *z* = −0.60; *p* = 0.550
Time estimation	TE1 (short): *U* = 53.0; *z* = −1.16; *p* = 0.247	TE2 (long): *U* = 43.0; *z* = −1.56; *p* = 0.120

RT: reaction times; PM: prospective memory; OGonly: performance in ongoing task trials in the phase where they performed alone (before a prospective memory instruction has been given); OGPM: performance in ongoing task trials in the phase where they are mixed with PM task; PM trials: only performance in prospective memory trials is considered.

* Performance accuracy in time-based PM trials refers to the average delay, calculated for each participant, between two space bar presses.

**Table 8:** ‘Left BA9 patients’ (from AnaCOM map of event-based tasks for pictures) compared to all ‘other patients’. Significant differences appear in bold font.

	OGonly	OGPM	PM
Time-based pictures			
Accuracy	*U* = 21.5; *z* = −1.87; *p* = 0.062	*U* = 45.0; *z* = −0.72; *p* = 0.474	*U* = 29.0; *z* = −0.22; *p* = 0.826*
RT	*U* = 58.0; *z* = −0.09; *p* = 0.924	*U* = 45.0; *z* = −0.72; *p* = 0.475	
Time-based words			
Accuracy	*U* = 38; *z* = −1.34; *p* = 0.182	*U* = 38.0; *z* = −1.05; *p* = 0.293	*U* = 29.0; *z* = −1.51; *p* = 0.880*
RT	*U* = 59.0; *z* = −0.05; *p* = 0.962	*U* = 58.0; *z* = −0.10; *p* = 0.924	
Event-based Pictures			
Accuracy	*U* = 56.0; *z* = −0.07; *p* = 0.791	*U* = 50.0; *z* = −0.54; *p* = 0.592	*U* = 49.5; *z* = −0.61; *p* = 0.545
RT	*U* = 60.0; *z* = −0.07; *p* = 0.944	*U* = 55.0; *z* = −0.30; *p* = 0.762	***U***** = 17.0;*****z***** = −2.00;*****p***** = 0.045**
Event-based words			
Accuracy	*U* = 26.0; *z* = −0.88; *p* = 0.377	*U* = 26.5; *z* = −0.67; *p* = 0.503	*U* = 31.0; *z* = −0.39; *p* = 0.700
RT	*U* = 28.0; *z* = −0.57; *p* = 0.567	*U* = 32.0; *z* = −0.32; *p* = 0.750	*U* = 13.0; *z* = −1.48; *p* = 0.140

Control tasks	Errors	RT
Simple RT		*U* = 47.0; *z* = −0.78; *p* = 0.436
Preparatory attending		*U* = 57.0; *z* = −0.27; *p* = 0.785
Target detection	*U* = 54.0; *z* = −0.70; *p* = 0.487	*U* = 36.0; *z* = −1.23; *p* = 0.219
Response inhibition	*U* = 55.0; *z* = −0.38; *p* = 0.702	*U* = 51.0; *z* = −0.55; *p* = 0.585
Multiple instruction	*U* = 59.0; *z* = −0.19; *p* = 0.852	*U* = 46.0; *z* = −0.77; *p* = 0.439
Task switching	*U* = 42.0; *z* = −1.17; *p* = 0.241	*U* = 40.0; *z* = −1.05; *p* = 0.295
Time estimation	TE1 (short): *U* = 59.0; *z* = −0.18; *p* = 0.856	TE2 (long): *U* = 62.0; *z* = −0.05; *p* = 0.964

RT: reaction times; PM: prospective memory; OGonly: performance in ongoing task trials in the phase where they performed alone (before a prospective memory instruction has been given); OGPM: performance in ongoing task trials in the phase where they are mixed with PM task; PM trials: only performance in prospective memory trials is considered.

* Performance accuracy in time-based PM trials refers to the average delay, calculated for each participant, between two space bar presses).

**Table 9:** ‘BA32/10 patients’ (from AnaCOM map of event-based tasks for words) compared to all ‘other patients’. Significant differences appear in bold font.

	OGonly	OGPM	PM
Time-based pictures			
Accuracy	*U* = 35.5; *z* = −1.81; *p* = 0.070	*U* = 51.0; *z* = −1.13; *p* = 0.258	***U***** = 5.0;*****z***** = −2.52;*****p*** = 0.012*
RT	*U* = 32.5; *z* = −1.92; *p* = 0.054	***U***** = 31.0;*****z***** = −1.97;*****p***** = 0.049**	
Time-based words			
Accuracy	*U* = 76.5; *z* = −0.08; *p* = 0.936	*U* = 37.5; *z* = −1.70; *p* = 0.090	*U* = 32.0; *z* = −0.81; *p* = 0.416*
RT	***U***** = 16.0;*****z***** = −2.59;*****p***** = 0.010**	***U***** = 27.0;*****z***** = −2.13;*****p***** = 0.033**	
Event-based Pictures			
Accuracy	*U* = 91.0; *z* = −0.25; *p* = 0.803	*U* = 84.5; *z* = −0.48; *p* = 0.630	*U* = 92.5; *z* = −0.20; *p* = 0.841
RT	***U***** = 31.0;*****z***** = −2.46;*****p***** = 0.014**	***U***** = 34.0;*****z***** = −2.35;*****p***** = 0.019**	*U* = 57.0; *z* = −1.32; *p* = 0.189
Event-based words			
Accuracy	*U* = 50.5; *z* = −1.83; *p* = 0.068	***U***** = 33.0;*****z***** = −2.19;*****p***** = 0.029**	*U* = 66.0; *z* = −0.81; *p* = 0.420
RT	***U***** = 15.0;*****z***** = −2.94;*****p***** = 0.003**	***U***** = 14.0;*****z***** = −2.98;*****p***** = 0.003**	*U* = 41.0; *z* = −1.73; *p* = 0.083

Control tasks	Errors	RT
Simple RT		***U***** = 17.0;*****z***** = −3.02;*****p***** = 0.003**
Preparatory attending		***U***** = 33.0;*****z***** = −2.42;*****p***** = 0.016**
Target detection	*U* = 70.0; *z* = −1.84; *p* = 0.066	*U* = 53.5; *z* = −1.68; *p* = 0.093
Response inhibition	*U* = 99.0; *z* = −0.04; *p* = 0.970	***U***** = 15.0;*****z***** = −3.07;*****p***** = 0.002**
Multiple instruction	*U* = 91.5; *z* = −0.32; *p* = 0.753	***U***** = 30.0;*****z***** = −2.43;*****p***** = 0.011**
Task switching	*U* = 88.0; *z* = −0.53; *p* = 0.595	***U***** = 44.0;*****z***** = −2.02;*****p***** = 0.043**
Time estimation	TE1 (short): *U* = 63.0; *z* = −1.34; *p* = 0.181	TE2 (long): ***U***** = 36.0;*****z***** = −2.31;*****p***** = 0.021**

RT: reaction times; PM: prospective memory; OGonly: performance in ongoing task trials in the phase where they performed alone (before a prospective memory instruction has been given); OGPM: performance in ongoing task trials in the phase where they are mixed with PM task; PM trials: only performance in prospective memory trials is considered.

* Performance accuracy in time-based PM trials refers to the average delay, calculated for each participant, between two space bar presses.
